# Unravelling Potential Health-Beneficial Properties of *Corema album* Phenolic Compounds: A Systematic Review

**DOI:** 10.3390/ph15101231

**Published:** 2022-10-08

**Authors:** Ana Sofia Cerquido, Martin Vojtek, Rita Ribeiro-Oliveira, Olga Viegas, Joana Beatriz Sousa, Isabel M. P. L. V. O. Ferreira, Carmen Diniz

**Affiliations:** 1 LAQV/REQUIMTE, Laboratory of Pharmacology, Department of Drug Sciences, Faculty of Pharmacy, University of Porto, 4050-313 Porto, Portugal; 2 LAQV/REQUIMTE, Laboratory of Bromatology and Hydrology, Department of Chemical Sciences, Faculty of Pharmacy, University of Porto, 4050-313 Porto, Portugal; 3 Faculty of Nutrition and Food Sciences, University of Porto, 4150-180 Porto, Portugal

**Keywords:** plant, natural products, camarinha, antioxidant, polyphenols, anticancer, anti-inflammatory

## Abstract

*Corema* (C.) *album* belongs to the family Ericaceae and can be found in the Iberian Peninsula, especially on the coastal areas facing the Atlantic coast. *C. album* berries have been used for centuries in traditional medicine. Recent studies have revealed that not only the berries but also the leaves have relevant antioxidant, antiproliferative, and anti-inflammatory properties, bringing this plant to the forefront of discussion. A systematic review of the literature was carried out to summarize the phenolic compounds and bioactive properties identified in *C. album* berries and leaves and to search for research gaps on this topic. The search was conducted in three electronic databases (PubMed, SCOPUS, and Web of Science) using PRISMA methodology. The inclusion criteria were the chemical compositions of the berries, leaves, or their extracts and their bioactive properties. The exclusion criteria were agronomic and archaeological research. The number of studies concerning phenolic compounds’ composition and the bioactive properties of *C. album* berries and leaves is still limited (11 articles). However, the variety of polyphenolic compounds identified make it possible to infer new insights into their putative mechanism of action towards the suppression of NF-kB transcription factor activation, the modulation of inflammatory mediators/enzymes, the induction of apoptosis, the modulation of mitogen activated protein kinase, cell cycle arrest, and the reduction of oxidative stress. These factors can be of major relevance concerning the future use of *C. album* as nutraceuticals, food supplements, or medicines. Nevertheless, more scientific evidence concerning *C. album’s* bioactivity is required.

## 1. Introduction

Plants as natural medicinal agents have been used since ancient civilizations to treat diseases such as cancer, inflammation, fever, etc. Their value as sources of molecules with therapeutic potential has been recognized and, recently, they have gained much attention in the drug discovery field, since many drugs from natural sources have been emerging, currently constituting up to 50% of all drugs in the pharmaceutical industry [1].

*Corema* belongs to the family Ericaceae and includes two species: *Corema conradii* and *Corema album*. The first is native to the Northwest coast of the USA and the latter can be found in the Iberian Peninsula, especially on the coastal areas facing the Atlantic [2]. In addition, *Corema conradii* differs from *Corema album* mainly by its very small fruit that lacks fleshiness and is covered with oily appendages [3]. Both species are coastal shrubs with sexual dimorphism. Concerning *Corema album*, this plant is a densely branched and long-living shrub with evergreen leaves [4]. While male flowers are bigger and have reddish petals and stamens with red-purple anthers, female flowers are smaller with pink-reddish petals [5]. Traditionally, the plant itself was also used in the Iberian Peninsula to make rustic brooms [6].

The berries from *Corema album* have been consumed for many centuries since the Islamic period (either fresh or in jams) and are employed in popular medicine [6,7]. In recent years, several reports have highlighted the health-beneficial properties of *Corema album* against several diseases, including cancer and neurodegenerative and cardiovascular diseases, and have ascribed most of their beneficial effects to their composition of phenolic compounds. Thus, this review is focused on unravelling the phenolic compounds already identified in *Corema album* with an emphasis on describing the biological mechanisms and signalling pathways related to their health-beneficial properties.

The species *Corema album* has two subspecies: *Corema album azoricum,* which is native to the islands of Azores, and *Corema album album* (*C. album*), which is more commonly found on the mainland [2]. The main difference between these two subspecies resides in its area of distribution: *Corema album azoricum* typically grows on volcanic lava or ash fields whereas *C. album* is characteristically found in coastal habitats [4].

*C. album* is an evergreen wild shrub that grows mainly on sandy soil over coastal dunes and cliffs, reaching a maximum height of 1 m, with numerous branches exhibiting leaves. Along the coast of Portugal, *C. album* is predominant on the southwest region from Sines to Troia and in the central-north region from Nazaré to Ovar [6]. The flowering of both female and male plants begins in early spring, from February to April [4]. The fruits are produced by the female plants and ripen in early summer (June and July) in the south and a little later (August and September) in the north [6]. The fruits are small, round berries coloured white or pink-white when ripe with an acidic flavour [8].

*C. album* has been used in traditional medicine and is one of the medicinal plants included in the herbarium of Francesc Bolòs (1773–1844) [9]. It has been described to exhibit beneficial properties against fever and intestinal pinworm infection [6,7], which is in accordance with the reported ability of *C. album* extracts to prevent oxidative damage [7]. Recently, the composition of each part of *C. album* has been studied by complementary Raman and infrared techniques, revealing vibrational signatures for the skin (outer and inner) and the seeds with distinct chemical compositions, specifically in its respective content in phenolic derivatives [10,11]. A systematic review of the literature was carried out to summarize the phenolic compounds and bioactive properties identified in *C. album* berries and leaves and to search for research gaps in this topic.

## 2. Methods

### 2.1. Search Strategy

PRISMA methodology was applied by performing a search for publications in three databases, namely, PubMed, SCOPUS, and Web of Science, using the following keywords: (“*Corema album*” AND (berries OR leaves)). The collection of papers was performed up to 15 June 2022. A total of 74 publications were identified after compiling all three databases. Duplicates, reviews, and opinion articles (n = 40) were removed.

### 2.2. Inclusion and Exclusion Criteria

The two authors of this publication independently screened the titles and abstracts of the 34 remaining articles. Inclusion criteria were studies focusing on chemical composition of berries, leaves, or their extracts and their bioactive properties. Exclusion criteria were agronomic and archaeological studies. Then, the full texts of eligible articles were carefully studied by all authors and the relevant data concerning phenolic compounds identified, *C. album* samples (berries, leaves, and extracts), and the bioactive properties studied were collected. In all steps, disagreements were resolved by meeting all authors and deciding on the inclusion or exclusion of the articles together.

## 3. Results

### 3.1. Literature Search Process

**Figure 1:**
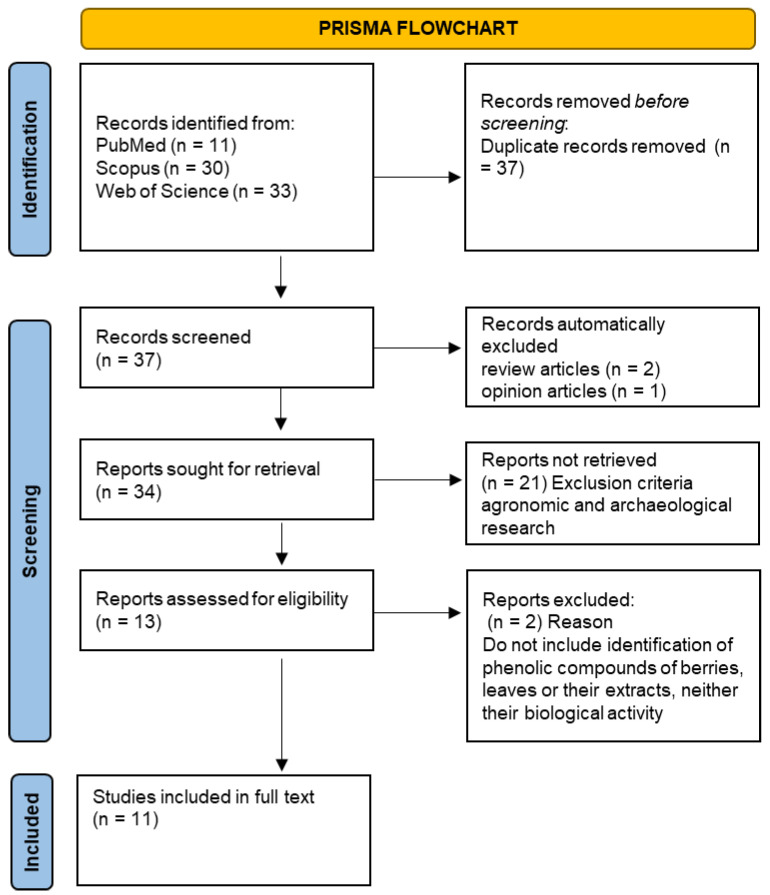
Flowchart summarizing the literature selection process according to PRISMA methodology.

From the 74 records identified, only 34 remained for the title and abstract screening. The remaining reports were duplicated articles, reviews, or opinion articles (Figure 1). Then, 21 articles were excluded based on the title and abstract reviews because these studies involved agronomic or archaeological studies and did not include the chemical composition of the berries, leaves, or of the respective extracts. The remaining 13 papers proceeded to the full text review. From those, only 11 were about phenolic compounds’ identification in *C. album*; thus, they were considered eligible for the data extraction [7,10,11,12,13,14,15,16,17,18,19].

### 3.2. Phenolic Compounds in Berries and Leaves from C. album

Both berries and leaves from *C. album* revealed a rich content in several phenolic compounds, which are summarized in Table 1 and Table 2, respectively. The phenolic compounds were divided into three main groups, namely, phenolic acids, flavonoids, and stilbenes, according to their structural similarities. Phenolic acids are commonly divided into two groups: the benzoic acids (C6-C1) with seven carbon atoms and cinnamic acids (C6-C3) with nine carbon atoms. Usually, these compounds occur predominantly in their hydroxylated forms: hydroxybenzoic and cinnamic acids, respectively. Flavonoids present a basic structure with 15 carbon atoms distributed by two aromatic rings linked by a three-carbon chain (C6-C3-C6). Stilbenes are known to display a structure with two aromatic rings linked by an ethene bridge.

Moreover, and in accordance with previously described features [20], natural phenolic acids, free or conjugated, can also appear as amides or esters whereas natural flavonoids, free or conjugated, are often esterified to one or two sugar molecules (by one or more hydroxyl groups).

**Figure 2:**
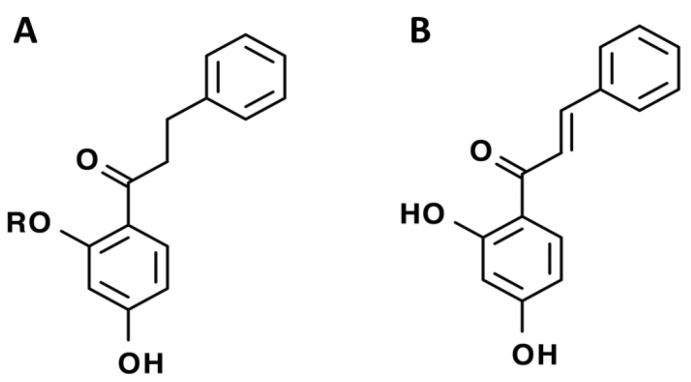
Structure of identified chalcones in *C. album*: (**A**) R = OH, 2′,4′-dihydroxydihydrochalcone, R = OCH_3_, 2′-methoxy-4′-hydroxydihydrochalcone; (**B**) 2′,4′-dihydroxychalcone.

In *C. album* leaves, another three predominant compounds were identified: 2′,4′-dihydroxydihydrochalcone, 2′-methoxy-4′-hydroxydihydrochalcone [17], and 2′,4′-dihydroxychalcone [18] (Figure 2). These compounds are chalcones, which are intermediates in the biosynthesis of flavonoids and isoflavonoids [19].

**Figure 3:**
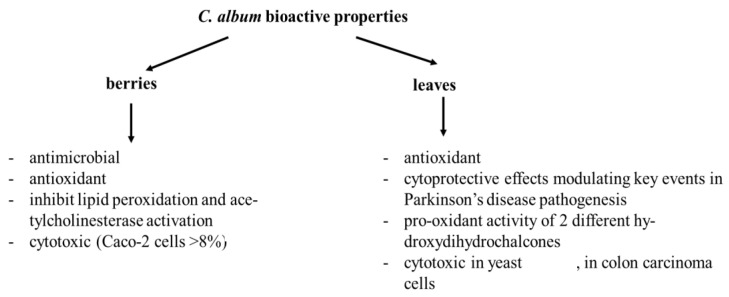
Summary of bioactive properties found in the literature for C. album berries, leaves, or their extracts.

Both the berries and leaves from *C. album* revealed interesting bioactive properties, which are summarized in Figure 3. Scientific information regarding *C. album’s* bioactive activities is very scarce and is mostly focused on the beneficial healthy properties of its berries. *C. album* berries were described to have antimicrobial [11] and antioxidant activities [7,11,14,15]. Moreover, this antioxidant activity seems to be increased after simulated digestion [13] and can protect against oxidative stress (yeast: [13]). *C. album* berries have also been described as having cytotoxic effects in Caco-2 cells when the concentration of the extract exceeds 8% [14]. There is also evidence that these berries are able to inhibit lipid peroxidation and acetylcholinesterase activation [11].

The bioactivity of *C. album* leaves has been studied regarding its cytotoxicity in yeast [15,20]; colon carcinoma cells (HT-29 cells: [17]), an effect that seems to be mediated through G2/M cell cycle arrest [18,21]; and apoptosis [18]. The cytotoxicity observed was reported to be triggered by the pro-oxidant activity of at least two different hydroxydihydrochalcones found in these leaves [17]. In contrast, a study using an enriched fraction of polyphenols from *C. album* leaves claimed that this extract has promising cytoprotective effects, modulating key events in Parkinson’s disease pathogenesis. Some other reports also describe *C. album* leaves as having antioxidant effects [15,20].

## 4. Discussion

A wide variety of phenolic compounds were identified in the berries and leaves from *C. album*, but few studies explore the biological activities and signalling events triggered by their extracts. Nevertheless, their physical–chemical profile and high phenolic content supports a potential market expansion [22]. In particular, their enriched composition in phenolic compounds, both in the berries and leaves, bring valuable insights into their putative mechanism of action. Currently, it is well accepted that phenolic compounds can modulate the activity of several enzymes, kinases, and transcriptional factors involved in the modulation of biological processes such as oxidative stress, inflammation, cell proliferation, apoptosis, and cell death [21,23]. In accordance, the phenolic compounds previously identified in *C. album* berries and leaves are known to present a modulatory capability in several signalling pathways, signal mediators or enzymes, and/or kinases (Table 3 and Table 4). Thus, these mechanisms can be indirectly associated with *C. album.*

Note that the phenolic compounds may exert their biological effects through signalling pathways separately or in a sequential way. Moreover, a putative crosstalk between these pathways should not be overlooked.

### 4.1. Suppression of NF-kB Transcription Factor Activation

The nuclear factor kappa-light-chain-enhancer of activated B-cells (NF-κB) is a transcription factor involved in the regulation of the expression of several genes that are associated with inflammation and carcinogenesis. NF-κB, in the cytosol, is inactive since it is bound to inhibitor kB (IkB) [83]. When IκB is phosphorylated, NF-κB is free to be translocated to the nucleus and can activate genes such as p53, Myc, and other cellular genes [21,83]. Present in *C. album* berries, neochlorogenic [37,39,40], *p*-hydroxybenzoic [24], and ferulic [28,29] acids seem to be able to inhibit NF-κB activation. In addition, several flavonols such as catechins [66,67,68,69], quercetin rhamnosyl hexoside [78,79,80], myricetin [74,75], procyanidins [72,73] and kaempherol hexoside [76], identified in *C. album* leaves have been shown to suppress NF-κB transcriptional activity and, thus, can prevent inflammation and carcinogenesis.

### 4.2. Modulation of Inflammatory Mediators/Enzymes

All the phenolic acids identified in *C. album* berries present anti-inflammatory properties since they inhibit the production of several interleukins (IL-1β and IL-6) and TNF-α (Table 3). The following activities were reported in the leaves of *C. album*: some polyphenols, such as catechins, were shown to inhibit IL-6, IL-12, and IL-1α; IL-1β, TNF-α production [67,68]; procyanidins, shown to inhibit IL-1β and TNF-α expression [72]; quercetin rhamnosyl hexoside, which decreased the expression of TNF-α, IL-1β, IL-6, and IL-17 [78,81,82]; kaempherol hexoside, which suppressed TNF-α, IL-1β, and IL-6 generation [77]; myricetin, which reduced TNF-α, IL-12, and IL-6 expression [74]; and rhamnetin, which reduced TNF-α, IL-1β, IL-6, and IL-8 generation [84]. In addition, other compounds were identified in the leaves such as chalcone derivatives that inhibited the production of cytokines [85]; isoliquiritigenin and butein that inhibited lipopolysaccharide (LPS)-induced inducible nitric oxide synthase (iNOS); and cyclooxygenase-2 (COX-2) expression [86], contributing to the modulation of inflammation.

In the inflammatory process, enzymes such as COX-2 and xanthine oxidase (XO) play a key role, and their levels of expression are modulated during the inflammation’s progression. The polyphenols identified in the berries of *C. album* were shown to be capable of suppressing/reducing the activity of XO and/or COX-2: through phenolic acids such as chlorogenic [37] or p-hydroxybenzoic [24] acids or by flavonols such as quercetin-3-O-hexoside [48].

### 4.3. Induction of Apoptosis

Apoptotic regulation involves numerous proteins such as families of p53, bcl-2-like protein 4 (BAX), and caspases [23]. Several flavonols identified in *C. album* berries seem to be able to induce apoptosis: anthocyanins, such as delphinidin-3-O-hexoside can induce apoptosis by modifying BAX, caspase 3, and Bcl-2 proteins [64]; quercetin-3-O-hexoside seems to be able to promote apoptosis, enhancing the expression of p53 and BAX proteins [46]; and kaempherol-3-O-hexoside was associated with the induction of apoptosis through the upregulation of caspase 3 and the downregulation of Bcl-2 [41,43,44]. Another flavonoid identified in *C. album* leaves, pinocembrin, was reported to be able to induce apoptosis in many different types of cancer cells [87].

### 4.4. Modulation of Mitogen Activated Protein Kinase

Since mitogen-activated protein kinase (MAPK) pathways are a convergent avenue involved in numerous biological processes, changes in MAPK activity are of utmost importance. *p*-coumaric acid has been demonstrated to have both antioxidant and anti-inflammatory properties since it is capable of preventing oxidative stress-induced apoptosis in human epithelial cells through the modulation of the MAPK signalling pathway [88]. Other phenolic acids identified in *C. album* berries, such as ferulic acid [28,29], p-hydroxybenzoic acid [24], and neochlorogenic acid [36,40], can also prevent MAPK activation. In the leaves, some polyphenols have also been reported to exert modulatory effects on MAPK pathways, including quercetin rhamnosyl hexoside [79] and procyanidins [73].

### 4.5. Cell Cycle Arrest

The deregulation of the cell cycle is associated with carcinogenesis and phenolic compounds are known to be capable of inhibiting, in a variety of cell types, different cell phases (G1, S, S/G2, and G2) [21,89]. *C. album* flavonols, identified in the berries, are capable of changing the cell cycle; kaempherol-O-hexoside causes cell cycle arrest at G2 [43,45] while rutin (a quercetin derivative) induces G2/M cell cycle arrest [54].

### 4.6. Zeduction of Oxidative Stress

The antioxidant properties ascribed to *C. album* seem to be mediated by an upregulation of glutathione and cellularly antioxidant enzymes, as well as by the suppression of reactive oxygen species (ROS) generation [90,91]. Indeed, berries usually exhibit an enriched content of phenolic compounds commonly associated with their high antioxidant properties [15]. Such properties are also exhibited by *C. album* berries since they have an anthocyanin content that can inhibit the intracellular content of ROS [63,64,65]. In these berries, gallic acid, chlorogenic acid derivatives, and flavonols have also been identified as having antioxidant properties [15,24,51,53].

Some compounds identified in the leaves of *C. album* also have antioxidant properties: myricetin derivatives [74,75], reported as a modulator of nitric oxide (NO) generation and of iNOS activity; stilbene derivatives [92]; and prenylated chalcone glycoside, which showed radical scavenging activity [93].

## 5. Conclusions and Future Perspectives

Although a wide variety of phenolic compounds have been identified in the berries and leaves from *C. album*, at the time of this review (15th Jun 2022), there are scarce scientific data regarding the potential health benefits exerted by *C. album*. Only nine studies have evaluated the biological properties of the berries, leaves, or respective extracts of this plant. Nevertheless, the discussion section evidences that their rich composition in phenolic compounds is promising when considering their health benefits and therapeutic potential. The phenolic compounds identified in *C. album* leaves and berries can modulate several pathophysiological processes, namely, inflammation, oxidative stress, carcinogenesis, etc., and this plant may also be attractive to the pharmaceutical industry with respect to generating new drug(s), nutraceuticals, or supplements, but more scientific evidence concerning *C. album’s* bioactivity is required.

## Figures and Tables

**Table 1:** Phenolic compounds identified in *C. album* Berries.

Group	Sub-Group	Compound	General Structure	Ref.
PHENOLIC ACIDS		Benzoic acid	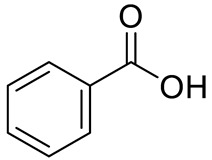	[12]
		Salicilic acid	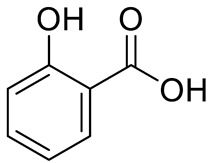	[12]
		Tannic acid	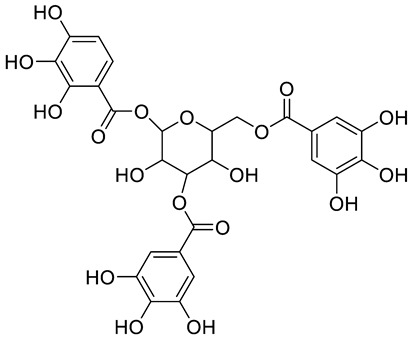	[13]
	Hydroxibenzoic acids	*p*-hydroxybenzoic acid (R=R_1_=R_2_=H) and derivatives Vanillic acid (R=R_1_=H; R_2_=OCH_3_) Protocatechuic acid (R=R_1_=H; R_2_=OH) Syringic aid (R=H; R_1_=R_2_= OCH_3_) Gallic acid (R=H; R_1_=R_2_=OH)	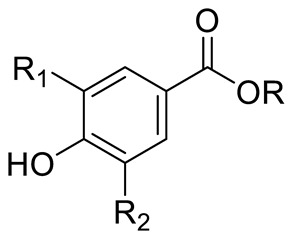	[7,10,11,12,14]
	Hydroxicinnamic acids	*t*-Cinnamic acid	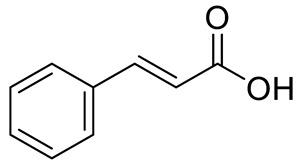	[12]
		*p*-coumaric acid (R=R_1_=R_2_=H) Sinapic acid (R=H; R_1_=R_2_=OCH_3_) Ferulic acid (R=R_2_=H; R_1_=OCH_3_) and derivatives Caffeic acid and derivatives (R=R_2_=H; R_1_=OH/O-Hexose; R_2_=H)	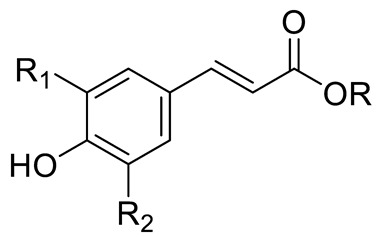	[7,12,14]
		Chlorogenic acid (R=R_1_=R_2_=H; R_3_=Caffeic acid) Neochlorogenic acid (R=R_1_=R_2_= H; R_3_= Caffeic acid) Cryptochlorogenic acid (R=R_2_=R_4_=H; R_3_= Caffeic acid)	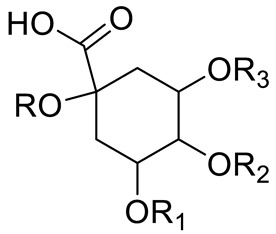	[7,14,15,16]
FLAVONOIDS	Flavonols	Kaempherol (R_1_=R_2_=R_4_=OH; R_3_=R_5_=R_6_=H) and derivatives: - i.e., Kaempherol 3-O-galactoside (R_6_=galactose) - i.e., Kaempherol 3-O-glucoside (R_6_=glucose)	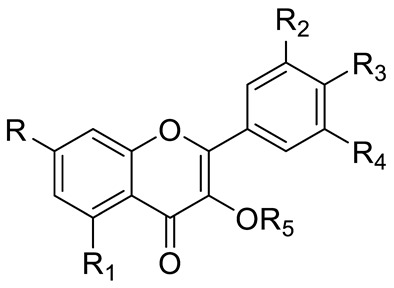	[7,14,15,16]
		Quercetin (R=R_1_=R_2_=R_3_=OH; R_4_=R_5_=H) and derivatives: - i.e., Quercetin 3-O-glucoside (R_5_=glucose) - i.e., Quercetin 3-O-arabinoside (R_5_=arabinose) - i.e., Quercetin 3-O-galactoside (R_5_=galactose) - i.e., Quercetin rhamnoside (R_5_=rhamnose) Rutin (R= R_1_= R_2_=R_3_=OH; R_4_=H; R_5_=glucopyranose)		[7,15,16]
		Myricetin (R=R_1_=R_2_=R_3_=OH; R_4_=H; R_5_=OH) and derivatives: - i.e., Myricetin 3-O-glucoside (R=R_1_=R_2_=R_3_=OH; R_4_=H; R_5_=O-glucose)		[7,15,16]
		Catechin (R=R_1_=R_2_=R_3_=R_4_=R_5_=OH) and derivatives		[15]
		Procyanidin (R=H, n=1) and derivatives: - i.e., Procyanidin Dimer type A (R=H, n=2)	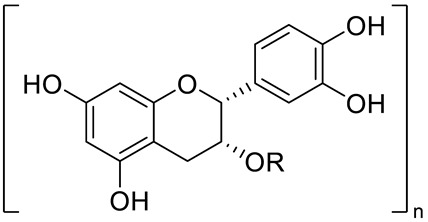	[15]
	Flavanones	pinocembrin	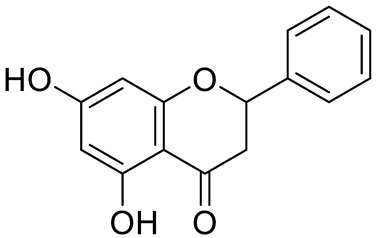	[7]
		6-geranylnaringenin	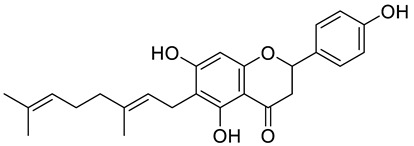	[7]
	Anthocyanins	Cyanidin (R_1_=R_2_=OH; R_3_=R_4_=R_5_=R_6_=H) and derivatives - i.e., Cyanidin 3-O-glucoside (R_6_=glucose) - i.e., Cyanidin 3-O-arabinoside (R_6_=arabinose)	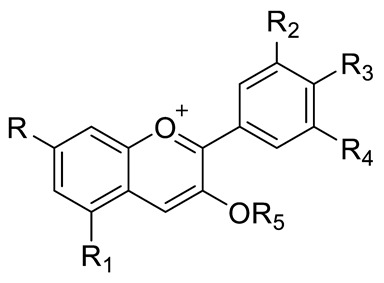	[7]
		Delphinidin (R_1_=R_2_=R_3_=R_4_=R_5_=OH; R_6_=H) and derivatives: - i.e., Delphinidin 3-O-glucoside (R_6_= glucose)		[7]
STILBENES		Resveratrol (R_1_=R_2_=H) and derivatives: - i.e., Pterostilbene (R_1_=R_2_=CH_3_) - i.e., Stilbene Hexoside (R_2_=Hexose)	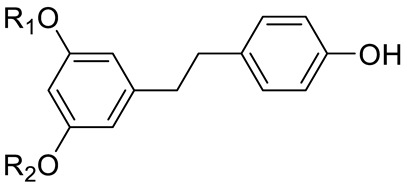	[7,15]

**Table 2:** Phenolic compounds identified in *C. album* leaves.

Group	Sub-Group	Compound	General Structure	Ref.
PHENOLIC ACIDS	Hydroxycinnamic acids	Coumaric acid (R=R_1_=R_2_=H) and derivatives: - i.e., Coumaroyl Glucose (R=Glucose)	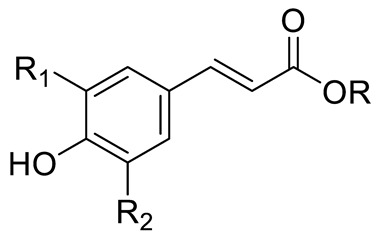	[15,16]
FLAVONOIDS	Flavanols	Catechin (R=R_1_=R_2_=R_3_=R_4_=R_5_=OH) and derivatives: - i.e., Catechin 3-O-glucose (R_3_=Glucose) Epicatechin (R=R_1_=R_2_=R_5_=OH; R_3_=R_4_=H)	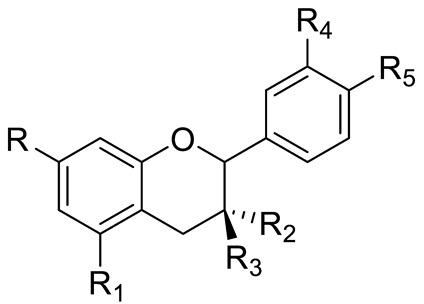	[15,16]
		Procyanidin (R=H, n=1) and derivatives: - i.e., Procyanidin Trimer (R=H, n=3) - i.e., Procyanidin Tretramer (R=H, n=4) - i.e., Procyanidin Galhate (R=Galhate, n=1)	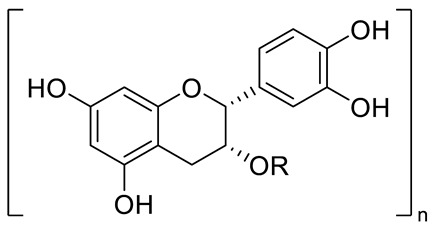	[15,16]
	Flavones or Flavonols	Myricetin (R=R_1_=R_2_=R_3_=R_5_=OH; R_4_=H) and derivatives: - i.e., Myricetin 3-O-galactoside (R_5_=O-galactose) - i.e., Myricetin 3-O-glucoside (R_5_=O-glucose) - i.e., Myricetin Xyloside (R_5_=O-xylose) - i.e., Myricetin Rhamnoside (R_5_=O-rhamnose) - i.e., Myricetin Methyl ether Hexoside	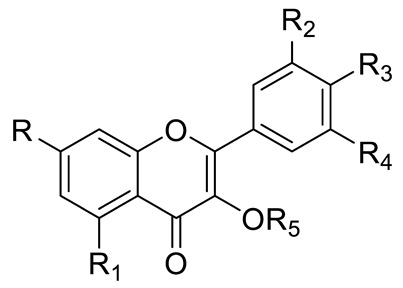	[15,16]
		Kaempherol (R=R_1_=R_3_=OH; R_2_=R_4_=R_5_=H) and derivatives: - i.e., Kaempherol Hexoside (R_5_=Hexose)	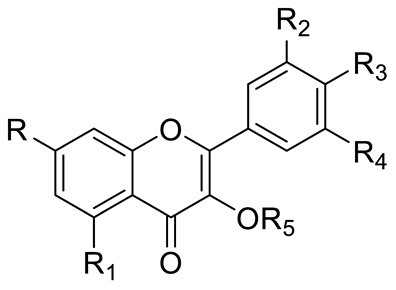	[15]
		Rhamnetin (R=OCH_3_; R_1_=R_2_=R_3_=OH; R_4_=R_5_=H) and derivatives: - i.e., Rhamnetin Hexoside (R_5_=Hexose)		[16]
		Quercetin (R=R_1_=R_2_=R_3_=OH; R_4_=R_5_=H) and derivatives: - i.e., Quercitin-3-O-glucoside (R_5_=glucose) - i.e., Quercitin-3-O-galactoside (R_5_=galactose) - i.e., Quercetin Rhamnosyl Hexoside (R_5_=Rhamnosoyl Hexose) - i.e., Methyl-quercitin hexoside (R_5_=Hexose) Rutin (R= R_1_= R_2_=R_3_=OH; R_4_=H; R_5_=glucopyranose)		[15,16]
		Pinocembrin	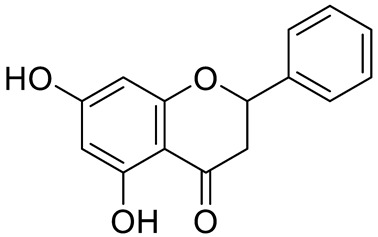	[18]
STILBENES		Stilbenes and derivatives	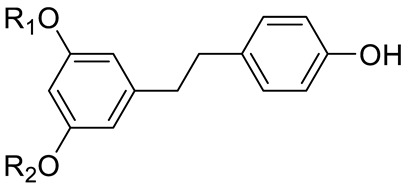	[15,16]

**Table 3:** Protective mechanisms ascribed to phenolic compounds identified in *C. album* berries.

Compound	Protective Mechanisms (s)	Experimental Model	Ref.
*p*-hydroxybenzoic acid	Inhibits iNOS/NO and COX-2/PGE_2_ production. Suppresses MAPKs, IKK, IkB, and p65 phosphorylation; and p65 nuclear translocation. Inhibits IL-1β, IL-6, and TNF-α production. Downregulates iNOS and COX-2 expression.	Mouse macrophages	[24]
*p*-coumaric acid	Suppresses apoptosis via modulation of MAPK signalling pathway. Suppresses IL-6 and TNF-α levels	- Human epithelial cells - Animal models: rheumatoid arthritis rats	[25,26]
Ferulic acid	Reduces UV-B radiation-induced oxidation. Suppresses NF-κB and MAPK pathways. Inhibits H_2_O_2_-induced MAPK activation via ROS pathway	- Human lymphocytes - Bovine endometrial epithelial cells - Rat vascular smooth muscle cells	[27,28,29]
Caffeic acid and derivatives	Reduces mRNA and protein synthesis of TNF-α, IL-6, IL-1β cytokines. Induces apoptosis.	- Human cancer cells fibrosarcoma - Animal model: albino mice (BALB/c)	[30,31]
Chlorogenic acid	Downregulates LPS-induced COX-2 up-expression. Inhibits PGE_2_, NF-κB, JNK/AP-1 signalling pathway activation. Inhibits production of TNF-α, IL-6, IL-1β, IFN-γ, MIP-1α.	- Mouse macrophages	[32,33,34,35,36]
Neochlorogenic acid	Reduces production of TNF-α, IL-1β, IL-6 and NO. Inhibits NF-κB activation and blocks MAPK signalling pathway phosphorylation. Increases HO-1 expression via AMPK/Nrf2 signalling pathway activation. Reduces CM-activated IκB/NFκB, STAT3 expression, and Akt/mTOR pathways.	- Human cancer cells: lung - Mouse cells: macrophages, microglia, fibroblasts	[37,38,39,40]
Kaempherol and derivatives	Upregulates caspase-3 activity. Induces apoptosis. Inhibits cell growth. Induces Cell-cycle arrest at G2/M	- Human cancer cells: brain, breast, stomach, liver, QBC939 (human cholangiocarcinoma) - HCCC9810 (mice) and (human)	[41,42,43,44,45]
Quercetin and derivatives Rutin	Increases apoptosis. Inhibits cell cycle progression. Inhibits P-glycoprotein expression. Upregulates p53 and BAX expression. Downregulates PI3K, PKC, COX-2 and ROS expression. Downregulates hypoxia-induced Nox4. Inhibits xanthine oxidase. Inhibits lipid peroxidation. Induces G2/M cell cycle arrest. Increases apoptosis.	- Human cancer cells: breast, liver - Human cancer cells: neuroblastoma - Animal models: Calf lung and muscle cells; Albino rats of Wistar strain.	[46,47,48,49,50] [51,52,53,54]
Myricetin and derivatives	Increases apoptosis through reduction in Bcl-2 and pro-caspase-3 levels and increase in BAX and cleaved caspase-3 levels. Decreases cell proliferation through stimulation of phosphorylation and degradation of YAP. Increases cell cycle arrest. Reduces metastasis.	- Human cancer cells: esophagus, ovary, and liver	[55,56,57,58]
Cyanidin and derivatives	Reduces cell proliferation. Reduces IL-3 and IL-4 by GATA-3 inhibition. Increases apoptosis. Decreases mucin 4 expression. Increases fatty acid oxidation and AMPK activity.	- Human cancer cells: breast, liver, colon, prostate and ovarian. - Animal model: Murine thymoma	[59,60,61,62]
Delphinidin and derivatives	Inhibits BAX and caspase 3. Increases Bcl-2 protein. Inhibits intracellular ROS generation and Nox1 protein. Normalizes the enzyme activity of SOD, CAT, GSH-PX and MDA levels via increase in nuclear Nrf2 protein. Increases NF-κB and Nrf2 pathways antioxidant response. Inhibits activation of PI3K/Akt/mTOR components and secretion of proinflammatory cytokines and chemokines.	- Human cells (normal): eye, keratinocytes - Transformed cell line: human chondrocyte	[63,64,65]

Abbreviations: AKT—Protein kinase B; AP-1—Activator protein 1; BAX—Bcl-2-like protein 4; Bcl-2—B-cell lymphoma-2; CAT—Catalase; CM—conditioned medium; COX-2—Cyclooxygease-2; GSH-PX—Glutathione peroxidase; H_2_O_2_—hydrogen peroxide; HO-1—Heme oxygenase 1; IFN-γ—Interferon γ; IL—Interleukin; iNOS—Inducible nitric oxide synthase; IκB—NF-κB inhibitor; IKK—IκB kinase; JNK—c-Jun N-terminal kinase; lncRNA-MALAT1—Long non-coding RNAs of metastasis associated lung adenocarcinoma transcript 1; LPS—Lipopolysaccharide; MAPK—Mitogen-activated protein kinase; MDA—Malondialdehyde; MIP-1α—Macrophage inflammatory protein-1; mRNA—messenger RNA (ribonucleic acid); mTOR—mammalian target of rapamycin; NF-κB—Nuclear factor kappa-light-chain-enhancer of activated B-cell; NO—Nitric oxide; Nox1—NADPH (nicotinamide adenine dinucleotide phosphate) oxidase 1; Nox4—NADPH (nicotinamide adenine dinucleotide phosphate) oxidase 4; Nrf2—Nuclear factor erythroid factor 2-related factor 2; p53—Tumor protein p53; p65—Nuclear translocation of p65 subunit of NF-κB and NF-κB DNA binding activity; PGE_2_—Prostaglandin E2; PI3K—Phosphatidylinositol-3-kinase; PKC—Protein kinase C; ROS—Reactive oxygen species; SOD—Superoxide dismutase; STAT3—Signal transducer and activator of transcription 3; TNF-α—Tumor necrosis factor α; UV-B—Ultraviolet B; YAP—Yes-associated protein.

**Table 4:** Protective mechanisms ascribed to phenolic compounds identified in *C. album* leaves.

Compound	Protective Mechanisms (s)	Experimental Model	Ref.
Catechin and derivatives Epicatechin	Inhibits NF-κB and AP-1. Inhibits “pro-oxidant” enzymes and induces antioxidant enzymes. Suppresses inflammatory factors including NF-κB, cytokines and adhesion molecules. Reduces IL-6, IL-12, IL-1α and IL-1β mRNA expression induced by TNF-α.	- Animal studies: mice and rats. - Animal model: experimental autoimmune myocarditis rats, mouse fibroblasts	[66,67,68,69]
Procyanidin and derivatives	Upregulates expression and activity of antioxidant enzymes via ERK, JNK and p38 MAPK pathways. Upregulates Nfr2 expression and activates Nfr2 antioxidant response element-mediated transcription via p38 MAPK and PI3K/Akt pathways. Downregulates mRNA expression of proinflammatory cytokines such as TNF-α, IL-1β and inflammatory molecules of COX-2. Upregulates mRNA expression of IL-10 Suppresses MAPK, AP-1 and NF-κB pathways.	- Human cancer cells: liver - Animal models: Rat liver, mouse macrophages	[70,71,72,73]
Myricetin and derivatives	Inhibits production of pro-inflammatory mediators (NO, iNOS, PGE_2_, and COX-2). Decreases NO, iNOS, TNF-α, IL-6 and IL-12 production. Decreases NF-κB activation (suppresses degradation of IκBα, nuclear translocation of p65 subunit of NF-κB and NF-κB DNA-binding activity). Attenuates phosphorylation of STAT1 and IFN-β production. Upregulates HO-1 expression through Nrf2 translocation.	- Animal model: mouse macrophage, diabetic cardiomyopathy mice	[74,75]
Kaempherol and derivatives	Suppresses NF-κB pathway by targeting protein-docking sites. Modulates expression of inflammatory cytokines (TNF-α, IL-6, IL-1β and PGE_2_). Modulates phosphorylation of IκBα and p65. Inhibits phosphorylation of p38, ERK and JNK	- Human cancer cells: leukemia Animal model: mouse macrophage	[76,77]
Quercetin and derivatives	Downregulates the expressions of iNOS and IFN-γ Attenuates NF-κB-mediated inflammation. (Scavenges ROS, necessary for NF-κB activation, or blocks TNF-α-dependent commencement of nuclear translocation of NF-κB) Suppresses MIP-1α-mediated migration/activation of macrophages through downregulation of CCR1/CCR5 production and inhibition of inflammatory signalling activation in macrophages. Inhibits MAPKs (ERK and JNK) and transcription factors (NF-κB and AP-1). Downregulates mRNA and protein levels of TNF-α, IL-1β, IL-6, iNOS and MIP-1α Downregulates microRNA 155 levels, inhibiting NF-κB activation. Reduces IL-1β, TNF-α, IL-17 and intercellular adhesion molecule 1 production	- Animal model: HFD-induced inflammatory mice, mouse macrophages, male C57BL/6 mice, periodontitis mice	[78,79,80,81,82]

Abbreviations: AKT—Protein kinase B; AP-1—Activator protein 1; CCR1—C-C chemokine receptor type 1; CCR5—C-C chemokine receptor type 5; COX-2—Cyclooxygease-2; ERK—Extracellular signal-regulated kinase; HO-1—Heme oxygenase 1; IFN-β—Interferon β; IFN-γ—Interferon γ; IL—Interleukin; iNOS—Inducible nitric oxide synthase; IκBα—NF-κB inhibitor α; IκB—NF-κB inhibitor; JNK—c-Jun N-terminal kinase; MAPK—Mitogen-activated protein kinase; MIP-1α—Macrophage inflammatory protein-1; mRNA—messenger RNA (ribonucleic acid); NF-κB—Nuclear factor kappa-light-chain-enhancer of activated B-cell; NO—Nitric oxide; Nrf2—Nuclear factor erythroid factor 2-related factor 2; p65—Nuclear translocation of p65 subunit of NF-κB and NF-κB DNA binding activity; PGE_2_—Prostaglandin E2; PI3K—Phosphatidylinositol-3-kinase; ROS—Reactive oxygen species; STAT1—Signal transducer and activator of transcription 1; TNF-α—Tumor necrosis factor α.

## Data Availability

Data sharing not applicable.
